# Quantitative assessment of HCC wash-out on CT is a predictor of early complete response to TACE

**DOI:** 10.1007/s00330-021-07792-2

**Published:** 2021-03-18

**Authors:** Marco Fronda, Andrea Doriguzzi Breatta, Marco Gatti, Marco Calandri, Claudio Maglia, Laura Bergamasco, Dorico Righi, Riccardo Faletti, Paolo Fonio

**Affiliations:** 1 Department of Surgical Sciences - Radiology Unit, A.O.U. Città della Salute e della Scienza di Torino, Via Genova 3, 10126 Turin, Italy; 2 Department of Surgical Sciences - Radiology Unit, University of Torino – A.O.U. Città della Salute e della Scienza di Torino, Via Genova 3, 10126 Turin, Italy; 3 Department of Oncology- Radiology Unit, University of Torino – A.O.U. San Luigi Gonzaga di Orbassano, Regione Gonzole 10, Turin, Italy; 4 Department of Surgical Sciences, University of Torino – A.O.U. Città della Salute e della Scienza di Torino, C.so Bramante 88, 10126 Turin, Italy; 5 Department of Diagnostic Imaging and Interventional Radiology - Vascular Radiology, A.O.U. Città della Salute e della Scienza di Torino, C.so Bramante 88, 10126 Turin, Italy

**Keywords:** Carcinoma, hepatocellular, Tomography, X-ray computed, Biomarkers, Therapeutic chemoembolization

## Abstract

**Objectives:**

To investigate the predictive value of four-phase contrast-enhanced CT (CECT) for early complete response (CR) to drug-eluting-bead transarterial chemoembolization (DEB-TACE), with a particular focus on the quantitatively assessed wash-in and wash-out.

**Methods:**

A retrospective analysis of preprocedural CECTs was performed for 129 HCC nodules consecutively subjected to DEB-TACE as first-line therapy. Lesion size, location, and margins were recorded. For the quantitative analysis, the following parameters were computed: contrast enhancement ratio (CER) and lesion-to-liver contrast ratio (LLC) as estimates of wash-in; absolute and relative wash-out (WO_abs_ and WO_rel_) and delayed percentage attenuation ratio (DPAR) as estimates of wash-out. The early radiological response of each lesion was assessed by the mRECIST criteria and dichotomized in CR versus others (partial response, stable disease, and progressive disease).

**Results:**

All quantitatively assessed wash-out variables had significantly higher rates for CR lesions (WO_abs_ *p* = 0.01, WO_rel_ *p* = 0.01, and DPAR *p = *0.00002). However, only DPAR demonstrated an acceptable discriminating ability, quantified by AUC = 0.80 (95% CI0.73–0.88). In particular, nodules with DPAR ≥ 120 showed an odds ratio of 3.3(1.5–7.2) for CR (*p* = 0.0026). When accompanied by smooth lesion margins, DPAR ≥ 120 lesions showed a 78% CR rate at first follow-up imaging. No significative association with CR was found for quantitative wash-in estimates (CER and LLC).

**Conclusions:**

Based on preprocedural CECT, the quantitative assessment of HCC wash-out is useful in predicting early CR after DEB-TACE. Among the different formulas for wash-out quantification, DPAR has the best discriminating ability. When associated, DPAR ≥ 120 and smooth lesion margins are related to relatively high CR rates.

**Key Points:**

*• A high wash-out rate, quantitatively assessed during preprocedural four-phase contrast-enhanced CT (CECT), is a favorable predictor for early radiological complete response of HCC to drug-eluting-bead chemoembolization (DEB-TACE).*

*• The arterial phase of CECT shows great dispersion of attenuation values among different lesions, even when a standardized protocol is used, limiting its usefulness for quantitative analyses.*

*• Among the different formulas used to quantify the wash-out rate (absolute wash-out, relative wash-out, and delayed percentage attenuation ratio), the latter (DPAR), based only on the delayed phase, is the most predictive (AUC = 0.80), showing a significant association with complete response for values above 120.*

## Introduction

Hepatocellular carcinoma (HCC) accounts for about 90% of primary liver cancers and its incidence is growing on a global scale [1]. In cirrhotic patients, non-invasive imaging-based diagnosis with contrast-enhanced multiphasic computed tomography (CECT), dynamic contrast-enhanced magnetic resonance (MR), or contrast-enhanced ultrasound (CEUS) is widely accepted for nodules ≥ 1 cm, based on the high pretest probability and the typical combination of hypervascularity in the arterial phase and wash-out on portal venous and/or delayed phase. [2].

Transarterial chemoembolization (TACE) is the most widely used treatment for unresectable HCC [3, 4] and, according to the Barcelona-Clinic Liver Cancer (BCLC) stage system, is recommended for patients with BCLC stage B.

Since TACE can also be used as a bridge to liver transplantation, or in a stage migration strategy [5] when transplantation, resection, or ablation is not possible, it is estimated that up to 40% of TACEs are performed in the early (BCLC-A) or, rarely, advanced (BCLC-C) stage [6, 7]. Drug-eluting bead TACE (DEB-TACE), performed using embolic microspheres as carriers for chemotherapeutic agents, is widely used since a randomized phase II trial [8] showed a significant reduction in liver toxicity and drug-related adverse events, associated with a non-significant trend of better antitumoral effect, in comparison to conventional lipiodol-based TACE (cTACE). Although repeated treatments are often needed, a radiologic CR after the first TACE session is a robust predictor of favorable outcome [9].

In view of an optimal treatment allocation, several studies tried to identify preoperative imaging-based predictive factors for good response to chemoembolization, mainly based on tumor size, growth rate, margins, location, and, especially, grade of hypervascularization in the arterial phase [10–14]. Up to approximately 30% of HCCs, however, lack a clear arterial phase hyper-enhancement [15, 16], including very early HCCs, poorly differentiated HCCs, and HCC nodules with small hypervascular foci [17, 18]. Furthermore, a clear hyper-enhancement may be sometimes missed due to mistiming or artifacts in the arterial phase [19].

In previous studies, the delayed phase demonstrated to improve the sensitivity of biphasic CT scans in a diagnostic setting, with an excellent interobserver agreement [20], and to be superior to the portal venous phase for the detection of tumor wash-out [21]. However, to the best of our knowledge, the wash-out rate in the delayed phase was never studied as a predictive factor for radiological response to TACE.

The aim of this study is to investigate the preprocedural multiphase CECT predictive factors of early CR to DEB-TACE, with a particular focus on the quantitatively assessed wash-in and wash-out grade.

## Materials and methods

### Study design

A monocentric historic cohort was reviewed, including 279 patients subjected to transarterial treatment as first-line treatment for HCC between January 2016 and December 2019. In all patients, the decision to treat had been assessed in a multidisciplinary round.

Inclusion criteria were (i) HCC diagnosed by pathologic assessment or non-invasive diagnosis criteria according to the European Association for the Study of the Liver-European Organization for Research and Treatment of Cancer (EASL-EORT) Practice Guidelines [1]; (ii) intermediate-stage HCC according to the BCLC staging system stage, defined as a tumor number > 3, tumor size > 3 cm, or a single tumor > 5 cm, with no vascular invasion or extrahepatic metastasis; (iii) early-stage HCC in patients not suitable for ablation, resection, or transplantation; (iv) Child-Pugh score ≤ B7; (v) Eastern Cooperative Oncology Group (ECOG) performance status 0.

**Fig. 1:**
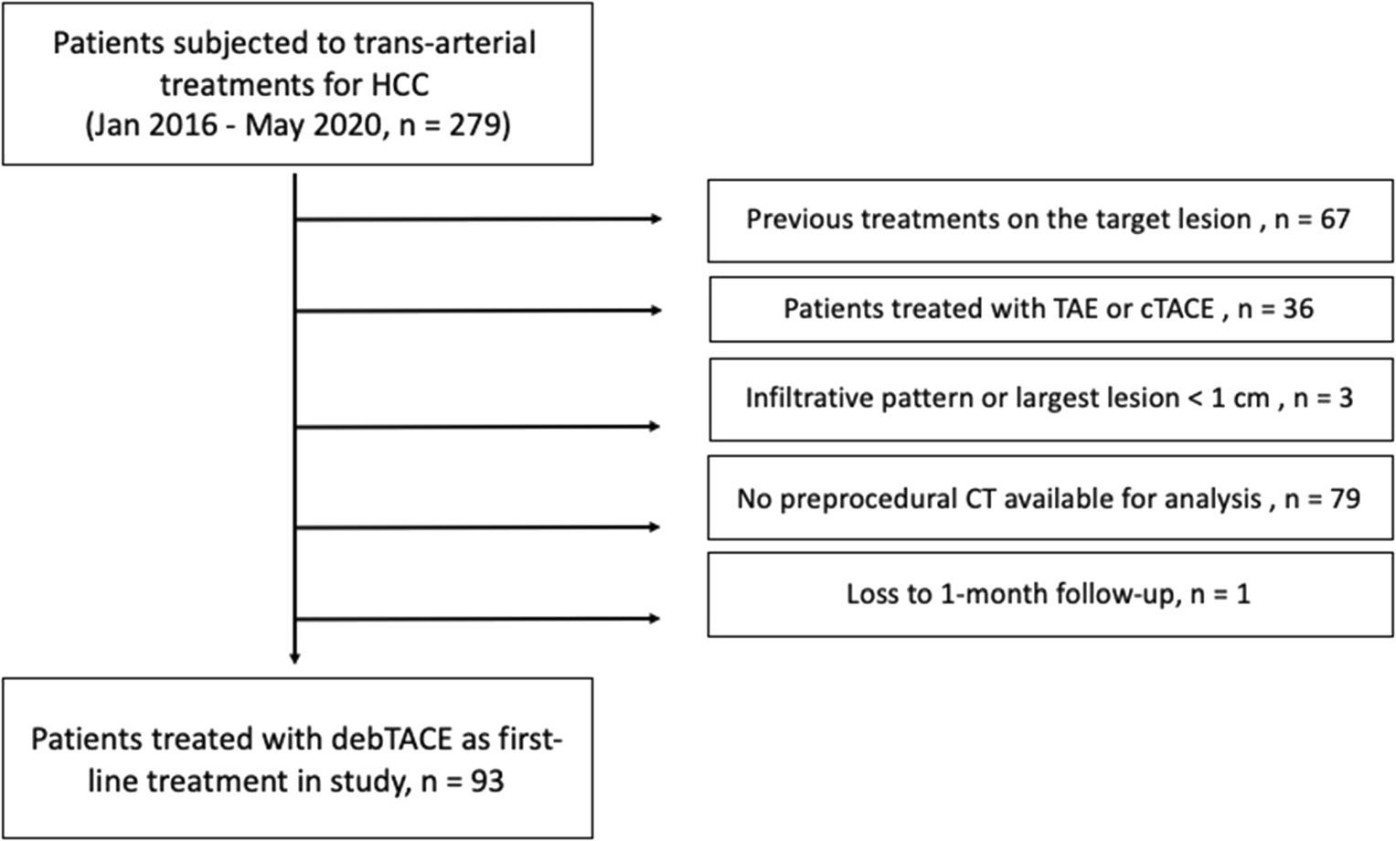
Study flow chart. Exclusion criteria

The exclusion criteria are shown in Fig. 1. Ninety-three patients satisfied all inclusion and exclusion criteria and were thus enrolled in the study.

The study is a retrospective trial without any study-related clinical intervention and conforms to the Helsinki Declaration. The study was approved by our Ethical Committee (protocol number 0098565). Before the TACE session, all patients were informed about the possible use of their data for study purposes. Patients’ information was anonymized before the analysis.

### Preoperative and follow-up imaging

According to the American Association for the Studies of Liver Diseases (AASLD) guidelines [22], multiphase CT was performed in the 2 months before the procedure and between 30 and 60 days after the procedure. All the CT scans were obtained by using a 64-slice CT scanner (Optima 660, GE Healthcare, *n* = 52) or a 256-row CT scanner (Revolution CT, GE Healthcare, *n* = 39). For dynamic studies, 1.5 mL/kg of iomeprol (Iomeron 400; Bracco) was injected at a rate of 4 mL/s, followed by a 40-mL saline flush injection at the same flow rate, using an automated injector (Stellant, Medrad).

CT scans were performed during the unenhanced phase, arterial phase, portal venous phase, and delayed phase. An automated bolus-tracking method (Snapshot Pulse/GE Healthcare) was used for the timing of the start of the arterial phase. A trigger threshold of an increase in the CT value of 100 HU (Hounsfield units) was placed in the descending aorta. Twenty seconds after exceeding the threshold level, the arterial phase scan was started. For the portal venous phase, there was a fixed 35-s delay following the end of the arterial phase and, for the delayed phase, a fixed 120-s delay following the end of the portal venous phase. Scanning parameters are shown in Table 1. For all CTs, post-processing reconstructions were performed on all native data in the transverse plane to yield 1.25-mm-thick sections.

**Table 1:** Preprocedural CECT. Scanning parameters of the two CT scanners

Scanner	Optima 660	Revolution CT
Pitch	0.984	0.984
Rotation time (s)	0.5	0.5
Slice thickness—acquisition (mm)	1.25	1.25
Detector configuration (rows × mm)	64 × 0.625	64 × 0.625
Tube voltage (kVp)	120	120
Tube current modulation (mA)	120–380 (NI 25)	120–380 (NI 19)

The follow-up CT scans were performed with the same protocol. Both images and radiologic reports were reviewed to determine the different responses on hepatic-arterial CT images by modified Response Evaluation Criteria In Solid Tumors (mRECIST) [23], as complete response (CR), partial response (PR), stable disease (SD), and progressive disease (PD).

### Preprocedural image analysis

**Fig. 2:**
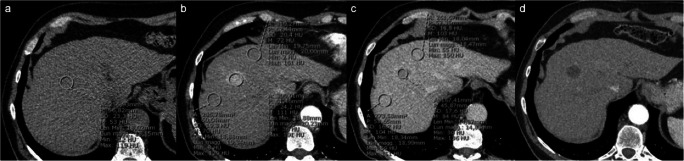
Tracing method of ROIs for the quantitative analysis. In order to compute CER, LLC, WO_abs_, WO_rel_, and DPAR, by each reader, one ROI was manually placed on the lesion in the unenhanced (**a**), arterial (**b**), and delayed (**c**) phase and two ROIs were placed on the adjacent hepatic parenchyma, excluding visible hepatic and portal vessels, bile ducts, and artifacts, both in the arterial (**b**) and delayed (**c**) phase. For the well-defined nodule showed in the example, the following quantitative parameters were calculated: CER 109.4, LLC 56.3, WO_abs_ 46.5, WO_rel_ 24.3, and DPAR 123.2. The 1-month follow-up CT scan shows CR, without any intratumoral arterial enhancement (**d**)

All initial CT images were downloaded using the picture archiving and communication system (PACS) of our center and analyzed in a blinded manner by two senior radiologists (M.F. and M.G.). For quantitative image analysis, an elliptic region of interest (ROI) was manually traced on the largest tumor area in the unenhanced, arterial, and delayed phase and two ROIs were placed on the adjacent hepatic parenchyma, excluding visible hepatic and portal vessels, bile ducts, and artifacts, in the arterial and delayed phase (Fig. 2).

The wash-in was quantitatively assessed as contrast enhancement ratio (CER) and lesion-to-liver contrast ratio (LLC) [24], the wash-out as absolute wash-out (WO_abs_), relative wash-out (WO_rel_), and delayed percentage attenuation ratio (DPAR), based on previous studies[25, 26].

In particular, the wash-in grade was quantitatively defined as:

$$ \mathrm{Contrast}\ \mathrm{enhancement}\ \mathrm{ratio}\ \left(\mathrm{CER}\right)=\left({\mathrm{HU}}_{\mathrm{arterial}}-{\mathrm{HU}}_{\mathrm{non}-\mathrm{contrast}}\right)/{\mathrm{HU}}_{\mathrm{non}-\mathrm{contrast}}\times 100 $$

$$ \mathrm{Lesion}-\mathrm{to}-\mathrm{liver}\ \mathrm{contrast}\ \mathrm{ratio}\ \left(\mathrm{LLC}\right)=\left({\mathrm{HU}}_{\mathrm{arterial}}-{\mathrm{HU}}_{\mathrm{liverart}}\right)/{\mathrm{HU}}_{\mathrm{liverart}}\times 100 $$

HU is the mean attenuation value of a ROI placed on the lesion in the arterial (HU_arterial_) or unenhanced (HU_non−contrast_) phase, or the average attenuation value of two ROIs placed in the healthy liver adjacent to the lesion in the arterial phase (HU_liverart_).

The wash-out grade was quantitatively defined as:

$$ \mathrm{Absolute}\ \mathrm{wash}-\mathrm{out}\ \left({\mathrm{WO}}_{\mathrm{abs}}\right)=\left[\left({\mathrm{HU}}_{\mathrm{arterial}}-{\mathrm{HU}}_{\mathrm{delayed}}\right)/\left({\mathrm{HU}}_{\mathrm{arterial}}-{\mathrm{HU}}_{\mathrm{non}-\mathrm{contrast}}\right)\right]\times 100 $$

$$ \mathrm{Relative}\ \mathrm{wash}-\mathrm{out}\ \left({\mathrm{WO}}_{\mathrm{rel}}\right)=\left({\mathrm{HU}}_{\mathrm{arterial}}-{\mathrm{HU}}_{\mathrm{delayed}}\right)/{\mathrm{HU}}_{\mathrm{arterial}}\times 100 $$

$$ \mathrm{Delayedpercentageattenuationratio}\left(\mathrm{DPAR}\right)={\mathrm{HU}}_{\mathrm{liverdel}}/{\mathrm{HU}}_{\mathrm{delayed}}\times 100 $$

HU is the mean attenuation value of a ROI placed on the lesion in the delayed phase (HU_delayed_), or the average attenuation value of two ROIs placed in the healthy liver adjacent to the lesion in the delayed phase (HU_liverdel_).

The qualitative evaluation of tumor margins was performed in a blinded manner on the delayed phase, according to a previous study [11], by the same two radiologists, and then dichotomized as smooth vs others (lobular or irregular).

The interobserver agreement was evaluated for all the analysis parameters.

### DEB-TACE procedure

Under local anesthesia, a 5-French (F) sheath was inserted in the right (*n* = 88) or left (*n* = 3) common femoral artery. Celiac axis and, if deemed necessary on the basis of preprocedural CT findings, superior mesenteric artery were catheterized with a 5-F catheter (Shepherd Hook II, TEMPO SHK1.0, Cordis).

An initial digital subtraction angiography (DSA) was performed with 20 mL of iopromide (Ultravist 370, Bayer Pharma) at a flow rate of 4 mL/s. Then, segmental or subsegmental feeding arteries of the hypervascular lesions identified were catheterized with a 2.7-F microcatheter (Progreat; Terumo) or, if necessary, 1.9-F microcatheter (Carnelian; Tokai Medical Product) with 0.014-in. microwire (Fathom; Boston Scientifics).

Procedures were carried out with DC beads (Biocompatibles) of different sizes ranging from 75–150 to 100–300 μm, following the recommendations of Biocompatibles (which varied during the study period) or, more recently, with LifePearl 100 ± 25 μm (Terumo), according to availability in our center. Bead loading was performed with a total amount of 50 mg of epirubicin (Farmorubicin, Pfizer). Once the microcatheter was in place, beads were diluted in 15 mL of contrast and saline mixed solution (in 50:50 mixed solution according to “Instructions for Use” recommendation) and administered manually under fluoroscopic guidance, in order to prevent reflux. The amount of the administered dose was adapted to reach complete devascularization of the target lesion and near stasis of the feeding vessel(s).

No other material was administered, except for Gelfoam (Gelatin sponge, Cutanplast, Mascia Brunelli S.p.A.) when intra-tumor bleeding was observed.

### Statistical analysis

Continuous variables that satisfied the Shapiro-Wilks normality test are expressed as a mean and standard deviation; otherwise, as median with first (Q1) and third (Q3) quartile. Categorical variables are presented as absolute numbers and percentages.

The univariate analysis used non-parametric tests: for 2 independent continuous variables, the Mann-Whitney test, for categorical variables Fisher’s exact test. Binary logistic regression (BLR) was run on variables determined as significant by the univariate analysis. Continuous variables with significant differences at BLR were dichotomized with the ROC curve procedure. The area under the curve (AUC) quantifies the ability of discriminating between two conditions, from 0.5 (chance) to 1 (excellent). The threshold was obtained through maximization of the harmonic mean of sensitivity and specificity and of Youden’s index and minimization of the distance of the curve from the upper left corner.

The statistical dispersion of a sample of quantitative data was estimated by the median absolute deviation (MAD), computed as the median of the absolute deviations of the data from their median.

The inter-reader agreement on quantitative variables was assessed with the non-parametric Wilcoxon’s test for matched data, the intraclass correlation coefficient ICC (0–1), and the *η*^2^ Cronbach’s coefficient (0–1), for qualitative evaluations with Cohen’s coefficient of concordance *k* (0–1).

Significance corresponded to *p < *0.05 and 95% CI of odds and risk ratios not including 1. The analysis was run on StatPlus: Mac v.7 (AnalystSoft).

## Results

### Patients’ sample and quantitative imaging analysis

The 93 patients enrolled in the study had an average age of 66 ± 11 years, 15(16%) women, all cirrhotic, 84 with Child class A and 9 with Child class B. The most present etiology was HCV (58%), followed by alcohol (18%) and HBV (15%). Sixty-nine patients had one lesion, 18 patients had two, 3 patients had three, 1 patient had four, 1 had five, and 1 had six, for a total number of 129 HCC lesions (1.4 per patient).

For the quantitative analysis of multiphase CECTs, a total of 903 ROIs were traced by each reader, as follows: 387 on the 129 tumors in the three different phases (mean size 1.40 cm^2^ ± 1.71 cm^2^) and 516 on the adjacent liver (258 ROIs in two different phases, mean size 1.44 cm^2^ ± 1.99 cm^2^).

**Fig. 3:**
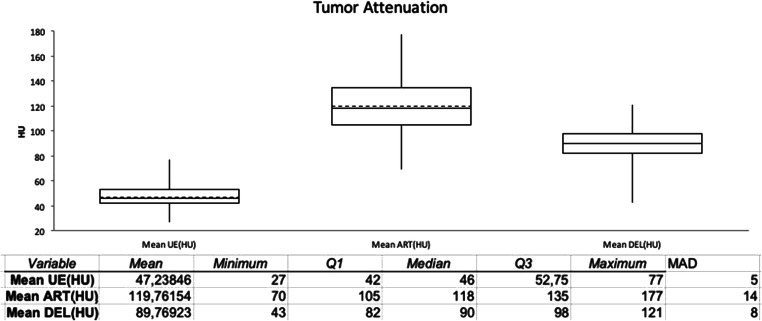
Boxplot of the attenuation values of the tumors in the unenhanced (UE), arterial (ART), and delayed (DEL) phase. The arterial phase showed the highest dispersion of the data set (median absolute deviation 14 vs 5 and 8 for the unenhanced and delayed phases, respectively)

Figure 3 is the boxplot of the attenuation values of the tumors in the unenhanced (mean UE), arterial (mean ART), and delayed (mean DEL) phase. The plot evidences the different dispersion of the three sets of data, quantified by the median absolute deviation (MAD) = 5 for the enhanced phase, 14 for the arterial phase, and 8 for the delayed phase.

The post-TACE evaluation of the tumor lesion response, performed according to the mRECIST criteria [23], evidenced 64 (49.6%) CR, 53 (41.1%) PR, 7 (5.4%) SD, and 5 (3.9%) PD. In the following, we shall consider CR as the primary predictor variable for prognostic factors for early radiological CR (CR+) after DEB-TACE.

### Identification of predictive factors

Table 2 compares the outcome variables of the preoperative multiphasic CECT for the 64 lesions with early radiologic CR+, with those of the other 65 lesions (CR-).

**Table 2:** Univariate analysis and binary logistic regression (BLR) of preoperative CECT parameters. CR+ vs CR- lesions

Outcome variable	Complete response (CR+), *N* = 64	No complete response (CR-), *N* = 65	Univariate *p* value	BLR *p* value	BLR odds ratio (95% CI)
Lesion location in liver					
Right	40 (61.5%)	41 (64.1%)			
Median	13 (20%)	10 (15.6%)	0.80		
Left	12 (18.5%)	13 (20.3%)			
Smooth margins	38 (59.4%)	20 (30.8%)	0.001	0.02	2.5 (1.1–5.5)
Maximum axial diameter (mm)	19 (14;25.5)	23 (16;33)	0.02	0.08	
Contrast enhancement ratio (CER)	150 (110;210)	150 (130;180)	0.98		
Lesion-to-liver contrast ratio (LLC)	70 (50;90)	70 (50;100)	0.74		
Absolute wash-out (WO_abs_)	46 (32;56)	36 (17;51)	0.01	0.10	
Relative wash-out (WO_rel_)	27 (18;35)	22 (10;32)	0.01	0.18	
DPAR	124 (116;139)	115 (108;123)	0.00002		
DPAR ≥ 120	41 (64%)	21 (32%)	0.0003	0.0026	3.3 (1.5–7.2)

The DEB-TACE outcome was not influenced by the size or type of beads used in the procedure (Table 3).

**Table 3:** Size of drug-eluting beads used for DEB-TACE procedures

	Complete response (CR), *N* = 64	No CR, *N *= 65	Univariate *p* value
DC beads 100–300 μm	36 (57%)	29 (45%)	0.25
DC beads 75–150 μm	13 (20%)	21 (32%)	0.17
LifePearl 100 ± 25 μm	15 (23%)	15 (23%)	0.87

The univariate analysis found that CR+ lesions had a significant higher incidence of regular margins than CR- (59% vs 31%, *p* = 0.001), smaller size (maximum axial diameter 19 (14;25.5) mm vs 23 (16;33) mm, *p* = 0.02), and higher quantitative wash-out variables. In particular, CR+ lesions showed significantly higher WO_abs_ (46 (32;56)% vs 36 (17;51)%, *p* = 0.01), WO_rel_ (27 (18;35)% vs 22 (10;32)%, *p* = 0.01), and DPAR (124 (116;139) vs 115 (108;123), *p* = 0.00002).

**Fig. 4:**
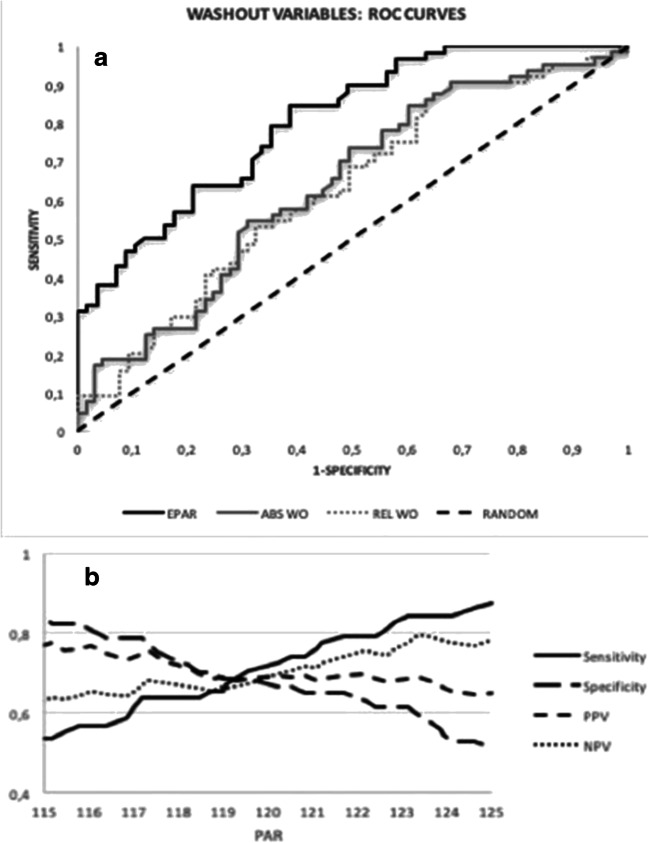
**a** Receiver operating characteristic (ROC) curves for WO_abs_, WO_rel_, and DPAR. For WO_abs_ and WO_rel_, AUC = 0.64 (95%CI 0.54–0.74) and 0.63 (95%CI 0.55–0.72) respectively; for DPAR, AUC = 0.80 (95%CI 0.73–0.88)**. b** Diagnostic parameters for DPAR in the 115–125 region including the threshold for complete response DPAR = 120

The ROC curve procedure used to measure the ability of the three parameters of wash-out (WO_abs_, WO_rel_, and DPAR) to discriminate between CR+ and CR- (Fig. 4a) yielded for WO_abs_ and WO_rel_ an AUC of around 0.60 with 95% CIs including 0.50, corresponding to a discrimination bordering on random. Conversely, for DPAR, AUC= 0.80 (95% CI 0.73–0.88), with DPAR ≥ 120 as the region significantly associated with CR. Figure 4b shows the diagnostic parameters in the region 115–125: for DPAR = 120, sensitivity 0.72, specificity 0.67, positive predictive value 0.69, negative predictive value 0.70, and diagnostic accuracy 0.70.

The multivariate binary logistic regression confirmed as significant predictor for CR+ only DPAR ≥ 120 (*p* = 0.003, OR= 3.3 (1.5–7.2)) and regular margins (*p* = 0.02; OR= 2.5 (1.1–5.5)).

For both parameters, the inter-reader agreement was very good: for DPAR, Wilcoxon’s *p=*0.98, ICC = 0.81 and *η*^2^ = 0.80; for margins, Cohen’s *k* = 0.76.

### Lesion stratification

The number of lesions with DPAR ≥ 120 was 62/129 (48%), with a presence of CR+ significantly higher than in the remaining 67 lesions: 41/62 (66%) vs 23 (34%), *p = *0.0004, OR = 3.7 (1.8;7.7). In the 33 cases in which the presence of DPAR ≥ 120 was accompanied by regular margins (25.6%), the presence of CR+ increased to 78.8% (26/33), *p* = 0.00002, OR = 3.2 (1.9;5.4).

**Fig. 5:**
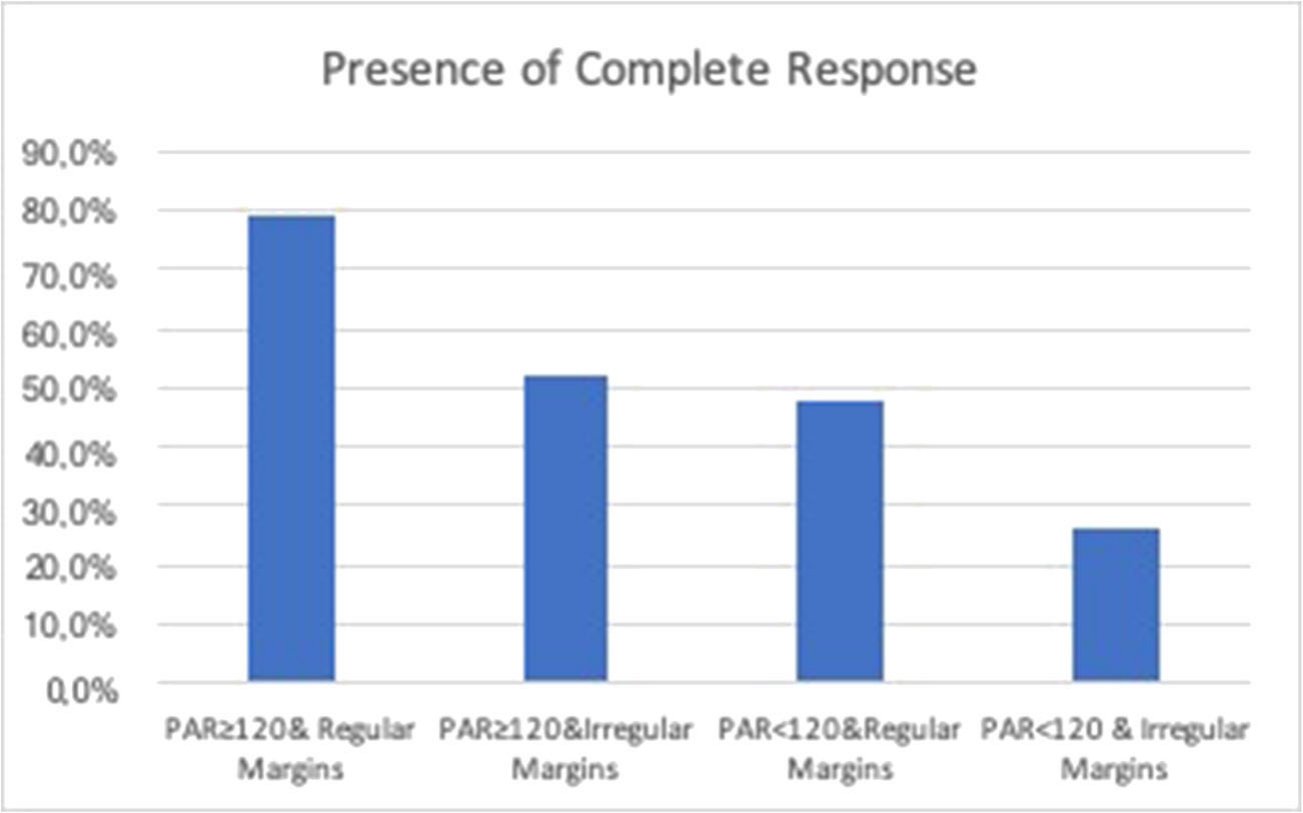
Lesion stratification for different preprocedural CECT outcomes. DPAR ≥ 120 and smooth margins showed a CR+ rate of 78.8% (26/33), while DPAR < 120 and non-smooth margins showed a CR+ rate of 26.2% (11/42) (*p* = 0.01)

**Fig. 6:**
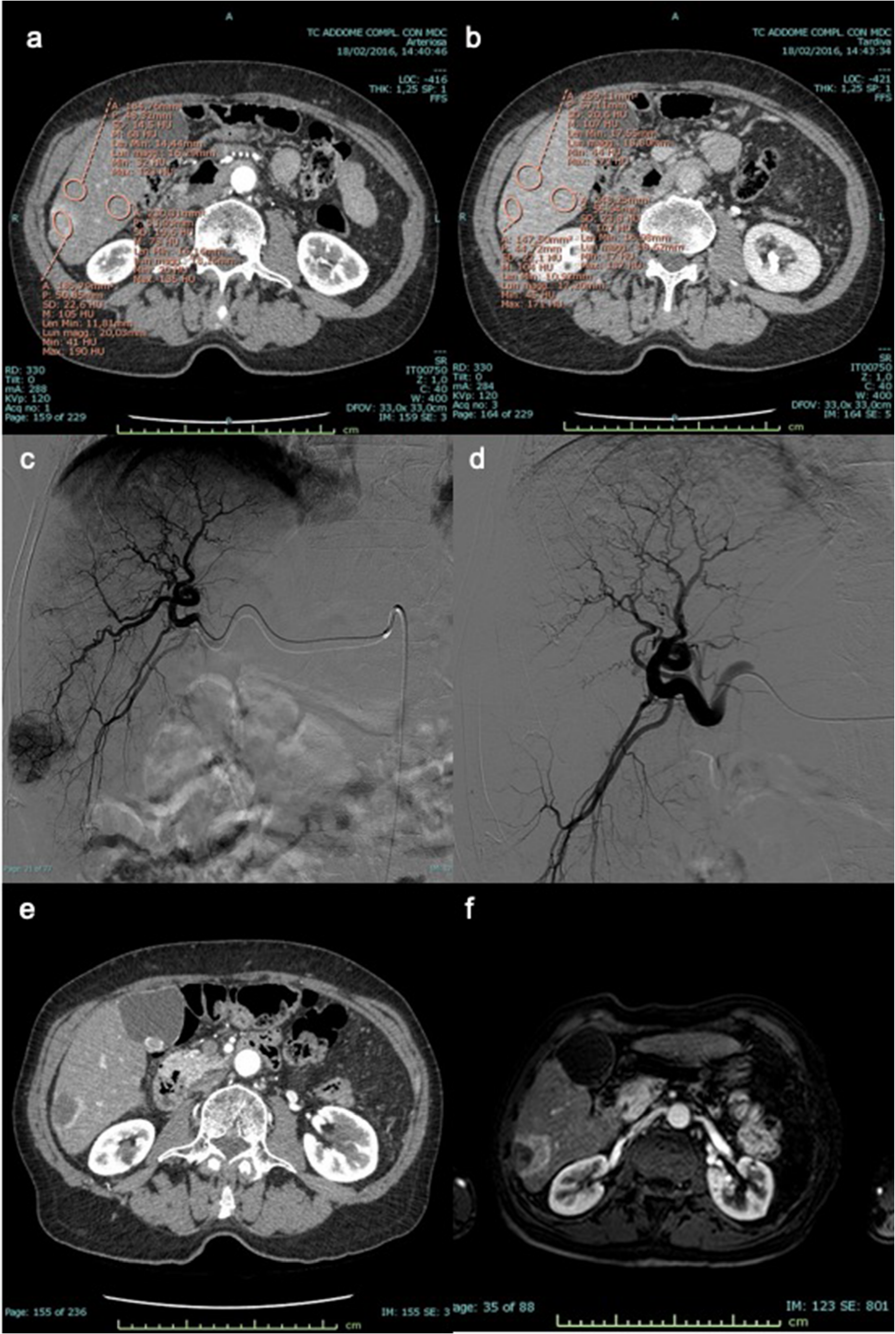
Preprocedural CECT, DSA, and follow-up cross-sectional imaging of an DPAR < 120 and non-smooth margin HCC nodule. For the HCC nodule showed in the example, the following quantitative parameters were calculated (**a**, **b**): CER 123.4, LLC 17.4, WO_ab_ 1.7, WO_rel_ 1, and DPAR 102.9. The preliminary DSA evidences a clear hyper-arterialization of the nodule (**c**), despite relatively low baseline CER and LLC values. While the final angiogram after DEB-TACE shows a complete devascularization of the nodule **(d**), the 1-month follow-up CECT evidences a residual crescent-shaped area of wash-in on the medial side of the scar (**e**). The 3-month follow-up MRI scan confirms the presence of obvious residual disease (**f**)

Figure 5 summarizes the CR+ rate to DEB-TACE for 4 possible preprocedural CECT outcomes: DPAR ≥ 120 and regular margins (CR+ rate 26/33 = 78.8%), DPAR ≥ 120 and irregular margins (CR+ rate 15/29 = 51.7%), DPAR < 120 and regular margins (CR+ rate 12/25 = 48%), and DPAR < 120 and irregular margins (CR+ rate 11/42 = 26.2%) (Fig. 6). The outcomes for the two cases of one favorable predictor (either DPAR ≥ 120 or smooth margins) are essentially similar (*p* ≥ 0.99), with an overall CR+ rate of 27/54 = 50%, significantly lower than for two favorable predictors (*p* = 0.01) and significantly higher than for no favorable predictors (*p* = 0.03). Lesions with two favorable predictors have a threefold higher chance to have a CR than lesions with none: *p* < 0.0001; RR = 3 (2–5).

## Discussion

The present study, performed on 129 HCC treated with DEB-TACE as first-line therapy, was aimed at investigating the prognostic value of the preoperative multiphasic CECT in predicting early radiological CR after DEB-TACE, with a particular focus on the quantitative assessment of wash-in and wash-out.

Our data showed an overall CR rate of 49.6%, in line with those reported in other per-lesion analyses [11, 13, 27]. Vesselle et al reported an overall CR rate of 26% [10]; it is to note, however, that their CR rate increased to 39% for lesions ≤ 5 cm. In our center, single large HCC nodules are generally subjected to combined therapy (thermoablation + TACE) rather than TACE; as a consequence, HCCs larger than 5 cm were only 3/129 (2.3%) in our cohort, making difficult a direct comparison with their study.

Several studies aimed to find predictive preoperative imaging biomarkers for early CR of HCC after chemoembolization. Our results confirmed the findings by Zhang et al [11] and Vesselle et al [10], about smooth margins and smaller size of the lesions being good predictors of higher susceptibility to TACE. In a previously published surgical series [28], HCC nodules without smooth margins showed a higher rate of histopathologic proven microvascular invasion which can explain, at least in part, their lower CR rate. The predictive value of size, we found with the univariate analysis (19 mm vs 23 mm for CR+ and CR- respectively, *p* = 0.02), was not confirmed by BLR, possibly because of the abovementioned scarcity of very large HCC nodules.

Unlike the study by Vesselle et al [10], in our cohort, tumor location in the so-called middle liver (ML, segments 1 and 4) was relatively rare (23/129, 17.8%) preventing the detection of a significant inverse relation with the radiological response.

Focusing on the quantitative assessment of lesions behavior during dynamic multiphase imaging, to the best of our knowledge, this is the first study in which a quantitative evaluation of the wash-out was systematically performed with predictive purposes.

In our study, CR+ lesions showed higher WO_abs_, WO_rel_, and DPAR. Among these, however, only DPAR showed a good discriminating ability (AUC = 0.80 (95% CI 0.73–0.88)) with a threshold value for CR located at DPAR ≥ 120.

The concurrent presence of well-defined margins and DPAR ≥ 120 resulted in 78% of radiological CR at the first follow-up imaging, much higher than what was generally reported for DEB-TACE. If confirmed on larger series, and with different CT equipment and protocols, this finding could be a useful tool to guide the treatment allocation of HCC patients.

These results are consistent with a previous study by Kwan et al [12], in which DPAR demonstrated to be the most reliable parameter to quantitatively assess wash-out and correctly identify pathologically proven HCC nodules in a diagnostic setting, with a cut-off value of 107.

In our opinion, these findings have their roots in the formulas used for calculation: both WO_abs_ and WO_rel_, in fact, employ the attenuation values of the tumors in the arterial (HU_arterial_) and delayed (HU_delayed_) phase, whereas DPAR is based only on the values in the delayed phase, which captures the lesions in their “steady state” during the dynamic multiphase study.

This hypothesis is supported by the significantly higher dispersion of the data set of the arterial phase compared to that of the delayed phase (MAD 14 vs 8, as shown in Fig. 3), which could also explain the null predictive value of the quantitative estimates for wash-in CER and LLC, despite some authors reporting a favorable predictive value of clear tumor wash-in when subjectively assessed during CECT [11] or preprocedural DSA [12, 29]. It is worth to note the fact that Zhang et al [11] evaluated the wash-in rate only in a qualitative way.

As regards DSA, it allows for a continuous evaluation of tumor hyper-arterialization, while CT captures the lesion in a single moment, with the possibility to miss the highest grade of hyper-enhancement, even with a standardized acquisition protocol.

Our hypothesis is that the wash-out rate may be interpreted as the downside of hyper-arterialization and, as a consequence of these previous findings, it might be used as a predictive factor for embolic therapies. Furthermore, in an extensive quantitative evaluation of the dynamic behavior of different HCC nodules in different CECT scans, the delayed phase, and then the DPAR, showed the lowest dispersion of the data set and the best predictive results.

If confirmed and externally validated, these findings could also help, in our opinion, laying the foundation to extend future artificial intelligence (AI) studies, to date mostly focused on the arterial phase [30, 31], to the delayed phase of multiphase CECTs.

This study has several limitations: (1) it is a retrospective single-center study; (2) large HCC nodules are very few in our cohort, and the applicability of the present findings to them needs to be investigated; (3) even if a radiologic CR after the first TACE session is known as a robust predictor of a favorable outcome, the impact of the predictive factors on survival was not assessed.

When quantitatively assessed during preoperative contrast-enhanced CT scan, wash-out is a better predictive biomarker than wash-in for early radiological CR of HCC to DEB-TACE.

Among the different ways to quantitatively evaluate the wash-out, the delayed percentage attenuation ratio (DPAR) showed the best predictive value. Smooth tumor margins and DPAR ≥ 120 are significantly associated with high CR rate after DEB-TACE.

## Abbreviations

BCLC: Barcelona-Clinic Liver Cancer
CECT: Contrast-enhanced multiphasic computed tomography
CER: Contrast enhancement ratio
CEUS: Contrast-enhanced ultrasound
DEB-TACE: Drug-eluting bead transarterial chemoembolization
DPAR: Delayed percentage attenuation ratio
EASL: European association for the Study of the Liver
ECOG: Eastern Cooperative Oncology Group
HCC: Hepatocellular carcinoma
LLC: Lesion-to-liver contrast ratio
MAD: Median absolute deviation
MR: Magnetic resonance
TAE: Transarterial embolization
WO_abs_: Absolute wash-out
WO_rel_: Relative wash-out
